# A community-based intervention study involving family gardens with aromatic herbs on changes in dietary and urinary sodium

**DOI:** 10.1186/s40795-024-00841-1

**Published:** 2024-02-26

**Authors:** M. Baston, M. Hernández-F, K. Vázquez, M. Ruiz-Morales, P. Mehner-Karam, M. Sil-Acosta, N. Acevedo, A. Granich-Armenta, K. Holz, A. Cantoral

**Affiliations:** 1 Health Department, Universidad Iberoamericana, Mexico City, Mexico; 2 Research Center for Equitable Development EQUIDE, Universidad Iberoamericana, Mexico City, Mexico; 3 Centro Ibero Meneses, Mexico City, Mexico; 4 Chemical, Industrial and Food Engineering Department, Universidad Iberoamericana, Mexico City, Mexico; 5 Instituto Nacional de Salud Pública, Cuernavaca, Mexico

**Keywords:** Family gardens, Aromatic herbs, Sodium, Home gardening, Workshops, Cooking

## Abstract

**Objective:**

To measure dietary and urinary changes in sodium (Na) intake and excretion through the implementation of family gardens with aromatic herbs and workshops for cooking, using the herbs as a substitute for salt and seasoning powder.

**Methods:**

Thirty-five participants from a neighborhood of Mexico City were included. A general questionnaire was administered to collect information on sociodemographic factors. At baseline and 3 months later, a dietary evaluation was conducted, and 24-hour urine samples were collected. Food items reported were classified according to the NOVA classification. Visits to participants´ houses were conducted to measure the amount of salt and seasoning powder added to food during the preparation of meals as well as a home food inventory. All participants were given a family garden with 6 aromatic herbs and a recipe book. The intervention included 7 cooking and 3 garden care workshops. Qualitative information on the experience was also collected. Linear regression models were run in order to estimate the contribution of each NOVA group, salt, and seasoning powder to total dietary Na intake.

**Results:**

Participants were 44 years old on average and were mainly women (91.4%). The participation compliance in the workshops was 69.5%. After 3 months, there was a Na intake mean reduction of 976 mg. There was also a reduction in the excreted urinary Na of 325 mg per day.

**Conclusion:**

A positive level of involvement in this program had a direct influence on dietary habits to lower Na consumption.

**Supplementary Information:**

The online version contains supplementary material available at 10.1186/s40795-024-00841-1.

## Introduction

Currently, 54% of the global population lives in urban areas. It is estimated that, due to increasing urbanization and significant population growth over 2.5 billion people will be living in cities by 2050, representing 68% of the total population living in urban zones [1].

This urban growth, combined with pollution, the nutrition transition, and the decrease of green spaces, has posed numerous challenges in the food and public health systems. Urbanization has significantly influenced dietary patterns [2]. The food industry has experienced exponential growth, introducing highly processed foods that are low in nutritional value, and high in calories and saturated fats, sodium, and sugars [3]. These, along with sedentary lifestyles, are the most significant risk factors contributing to the increasing prevalence and burden of overweight and obesity, type 2 diabetes, and cardiovascular diseases [3].

The sodium content in foods has been increasing. While sodium is an essential mineral for the human body, as it regulates blood pressure, blood volume, muscular contraction, nerve impulse transmission, acid-base balance, among other vital functions, excessive consumption can lead to elevated blood pressure, increasing the risk of cardiovascular diseases (the leading cause of death globally and in Mexico) [4], highlighting ischemic disease and stroke. Additionally, excessive sodium intake is associated with chronic kidney disease, obesity, gastric cancer, liver diseases, stroke, dementia, and disability [5]. According to the Global Burden of the Disease (2019), 23% of total deaths in Mexico were due to cardiovascular diseases, of which 5.4% were attributed to diet habits such as high sodium intake [6].

Worldwide, it is estimated that average sodium intake levels exceed the recommendations established by the World Health Organization (WHO), which are less than 2 g of sodium per day (equivalent to 5 g of salt) [5]. Therefore, one of WHO’s goals is to reduce the population’s average sodium intake by 30% by 2025 [7]. Different interventions in Mexico have been implemented to reduce sodium consumption. Some of these initiatives include removing saltshakers from restaurant tables, reducing sodium content by 10% in bread [8, 9], and implementing the new front-of-package labeling system for processed foods through NOM-051-SCFI/SSA1-2010 [10]. However, no studies have been conducted on the implementation of aromatic herb gardens to replace the use of salt and seasoning powder while cooking, even though it accounts for approximately 36% of the total sodium intake [11].

Family gardens in urban areas serve as a viable solution to counteract the negative effects of the modern diet by enabling the production and consumption of healthy, non-processed, and diverse foods. They offer benefits not only for physical health but also for social and emotional well-being [12]. Therefore, the aim of this study is to adapt and implement family gardens to generate a positive impact on dietary habits among individuals and their families.

## Methods

**Fig. 1:**
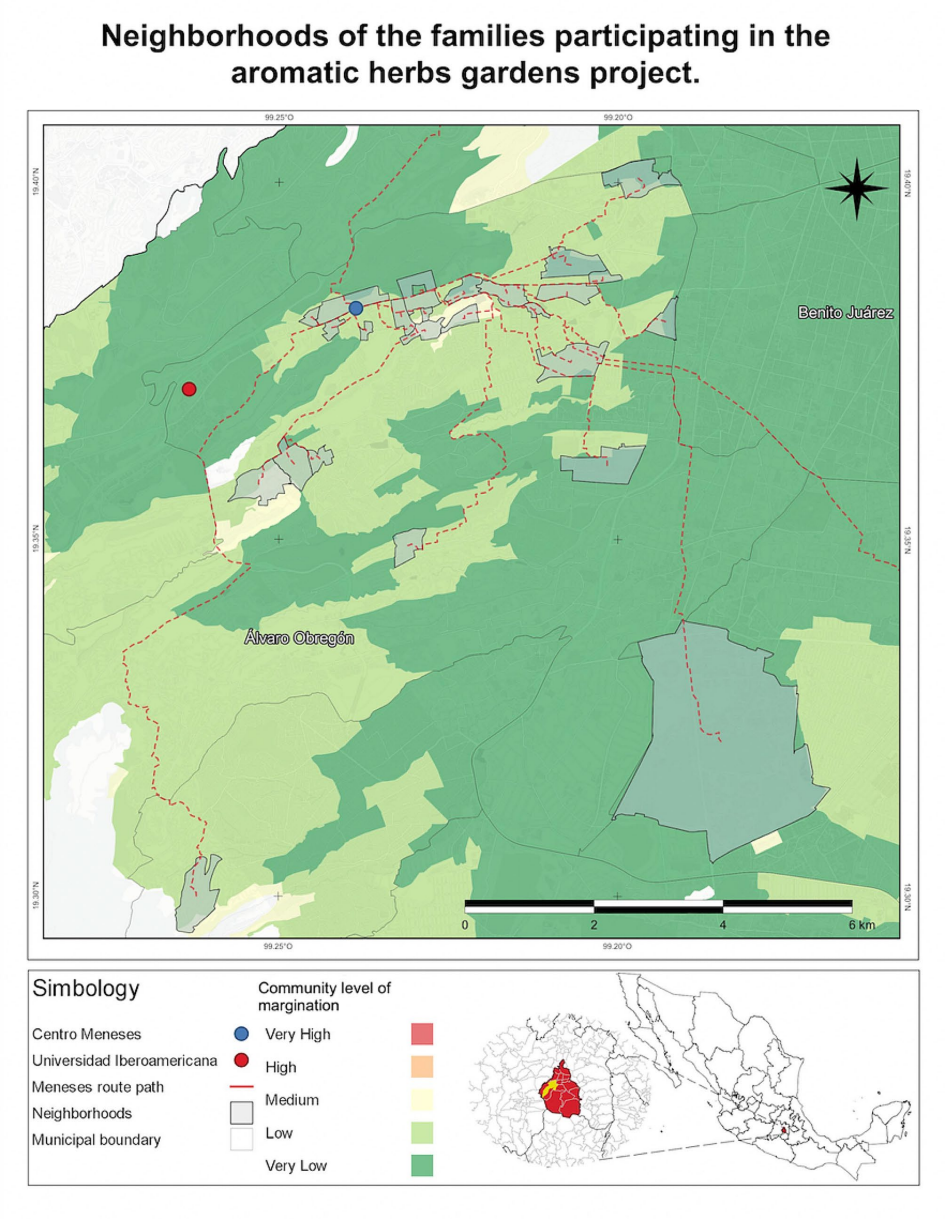
Neighborhoods of the families with regards to the distance to Meneses Center and Iberoamericana University

This is a community project performed in the neighborhood of Santa Fe, Mexico City. It was conducted from January 2022 to June 2022. The sample was selected using a convenience sampling method. The targeted population belonged to a low socioeconomic status, who live close to the Community Center “Centro Ibero Meneses” (Fig. 1), which is situated in an underserved area. This approach was chosen for its practicality and ease of access to participants. By focusing on families near the community center, the study aimed to ensure a representative sample from the local population in Santa Fe. Upon arrival at the Community Center, participants received a complete explanation of the program, and the head of the family was asked to provide informed consent to participate in this study. Subsequently, participants were asked to complete three distinct questionnaires, each serving a specific purpose and gathering diverse information related to sodium intake and family habits.

### General questionnaire

The general questionnaire was administered to one member from each of the 35 families in order to gather information about their sociodemographic and socioeconomic characteristics. The questionnaire included inquiries about who cooks the meals, what ingredients are used, the size and composition of their family, the age of each family member, the number of individuals who eat at home, and details about who is responsible for purchasing ingredients and foods, where the foods are bought (such as supermarkets, local markets, wet markets, small neighborhood stores), and how often they go shopping for food. Additionally, the questionnaire included questions about the participants’ knowledge of the relationship of sodium and health and whether anyone in their family lives with a non-communicable disease such as hypertension, type 2 diabetes, or overweight/obesity.

### 24-hour dietary recall

To collect 24-hour multi-step recall data, the ASA24 tool developed by the National Cancer Institute (NCI) was utilized. This automated website allows for the detailed capture of food and beverage consumption within the last 24 h, from midnight of the application day to midnight of the previous day. The project started by registering all participant folios in the ASA24 web system. Subsequently, the ASA24 tool was applied to participants via telephone, following a series of steps. This questionnaire allows participants to understand and describe their dietary habits and nutrient intake.

After collecting the participants’ diet data through the 24-hour recall, foods and food products were classified using the NOVA classification system, which groups them based on the extent and purpose of processing and consists of four groups [13]. Group 1 includes unprocessed or minimally processed foods, which are natural foods altered only through processes like drying or cooking. Group 2 comprises processed culinary ingredients obtained from foods or nature like salt and sugar. Group 3 consists of processed foods made by adding substances like sugar or salt to Group 1 foods. Finally, Group 4 includes ultra-processed products, which are industrial formulations with numerous ingredients added to processed substances, including additives [13].

### Sodium excretion in 24-hour urine

After administering the questionnaires, participants were shown a detailed video explaining how the 24-hour urine samples needed to be collected. They were then provided with sterilized containers to collect their urine samples. To determine the levels of sodium (Na) and potassium (K) in the urine, the samples were analyzed with the method of reflectance spectrophotometry. This technique is based on studying the behavior of electromagnetic waves emitted, absorbed, and reflected by a solid, liquid, or gas. It is commonly used for the identification of certain compounds and minerals such as Na and K [14].

### Family garden with aromatic herbs

**Fig. 2:**
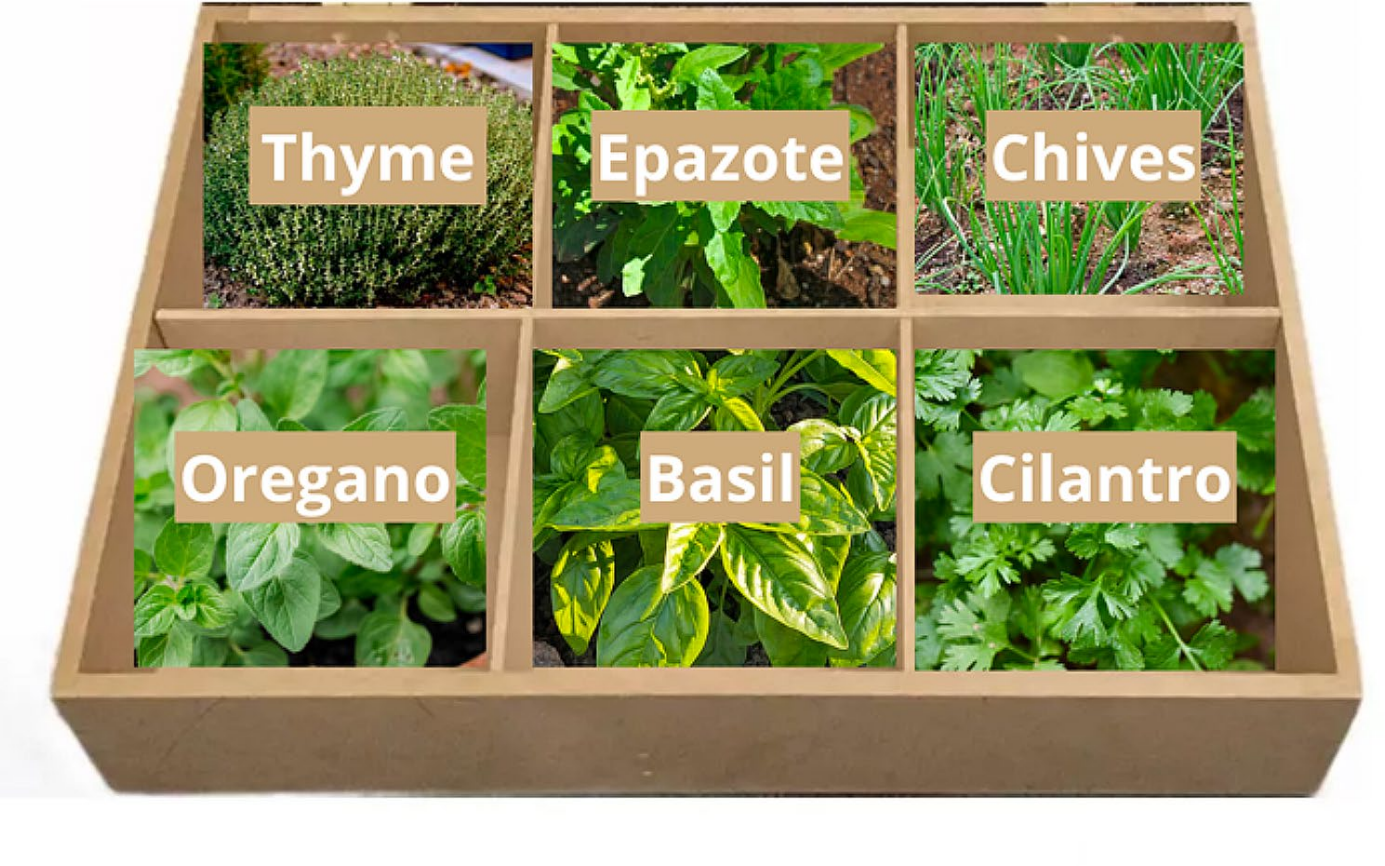
Family Garden illustration

Traditional Mexican cuisine is known for its use of various spices, condiments, and herbs to enhance the flavors of dishes [15]. As a result, a selection of herbs that can be used as alternatives to salt or seasoning powder were identified. Eight herbs, including parsley, oregano, chives, rosemary, cilantro, thyme, fennel, and basil, have been recommended as salt replacements [16]. These herbs not only add flavor but also stimulate the senses and digestive glands, contributing to better assimilation. They are used to enhance aromas, add spiciness and color to dishes, and some may even have antioxidant properties. With the experience of researchers working at “Huerto Ibero” a family garden with six herbs was designed based on adaptable plants for the microclimates of Mexico City, including cilantro, oregano, thyme, epazote, chives, and basil, in containers using a specific substrate mixture (Fig. 2). This family garden was given to each participant which helped them to harvest fresh herbs that can enhance the flavors and taste of Mexican dishes. Each garden had a cost of $300 Mexican pesos (around $15 US dollars).

### Workshops

Participants were given 7 cooking workshops via Zoom Video Communications Inc. (Zoom), each focused on preparing a dish using one of the herbs from the garden. The first workshop focused on the general introduction to the project and the cooking sessions, where a video was played explaining the importance of sodium, its sources, and the consequences of excessive consumption (Video 1). The subsequent workshops featured specific recipes, starting with a bean salad with basil vinaigrette and a pamphlet highlighting the nutritional properties of beans and basil, as well as a presentation on the new labeling regulations introduced in NOM-051 [10]. The second recipe was cilantro chicken, followed by esquites (a Mexican corn dish) with epazote, including information on the benefits of corn and epazote. The fifth workshop focused on making Chinese-style rice with chives, along with a session on proper food disinfection and the properties of eggs and chives. The second to last workshop featured celery soup with oregano, highlighting the properties of oregano and celery. Finally, the last recipe was a thyme-infused chicken, along with a pamphlet with information about the properties of thyme.

Lastly, they received 3 workshops also via Zoom on garden care, including topics such as selecting the appropriate soil for planting, understanding the different parts of a plant, where to find seeds, determining the spacing requirements for each plant, transplanting plants as they grow, watering and pruning frequency, knowing when the herbs are ready to be used, and addressing pest issues that may arise, among other subjects.

### Cooking recipe book

At the end of the workshops, participants were given a cookbook containing over 300 recipes created by four independent nutritionists specifically for the project. These recipes featured reduced sodium content of typical Mexican dishes, with sodium being replaced by various herbs from the family garden. The cookbook included a wide range of recipes, including beverages, appetizers, main courses, sauces and dressings, desserts, basic culinary techniques, and information about cooking methods, properties of herbs and spices, as well as infographics providing tips on reducing sodium consumption and understanding the sources of salt in our diets. The recipes used during the cooking workshops were sourced from this cookbook. The cost of printing each recipe book was $250 Mexican pesos (around $12.50 US dollars).

### Involvement with the families

There was constant communication with the families to answer any questions or concerns related to the intervention, providing information to them by an expert on garden care, three nutritionists, and the main researcher of the project. They were encouraged to ask any questions they had and were given the freedom to share recipes using aromatic herbs, as well as photos, videos or media of the dishes they prepared in the workshops and the growth and care of their own family garden.

### Direct measurement

Direct measurement involved quantifying the amount of salt and/or seasoning powder added to cooking in the households [4]. Each family household was visited and the person responsible for food preparation was asked to show the amount of salt and/or seasoning powder added to the meal on daily basis. The amount used was weighed using the Ohaus Scout Pro electronic scale and divided among the family members to determine individual sodium consumption.

For the further households from the Community Center, an online questionnaire was sent to request the quantity and frequency of salt and/or seasoning powder purchased. The amount purchased was then divided by the number of days between purchases and further divided by the number of family members. For example, if they purchased 1 kg of salt every 6 months, it would be divided as follows: 1000 g / 180 days by family = 5.5 g / 4 family members = 1.38 g of salt per person per day [4].

### Home food inventory

A validated instrument from the United States for Spanish-speaking families was used, which includes a list of common foods, beverages, and products found in the Mexican population’s pantry, refrigerator, and freezer. This instrument has shown that food availability in the household is significantly associated with dietary practices, intake, and eating patterns [17].

The qualitative data of the project was gathered through the storytelling method. It is an inherent element of human culture, and it has emerged as a valuable tool for gathering research data and developing multidisciplinary interventions [18].

It is important to highlight that due to the COVID-19 pandemic, some activities were adapted to an online format, utilizing platforms such as Zoom for conducting the workshops. Additionally, certain questionnaires, such as the 24-hour dietary recall, were administered via telephone to ensure the safety of the participants and collaborators.

In this study, for the purpose of translation and ensuring grammatical accuracy, the ChatGPT language model was utilized. ChatGPT was employed to aid in translating and refining specific paragraphs of the paper. Its advanced language capabilities were harnessed to enhance the clarity and coherence of the content, ensuring that the ideas and concepts presented maintain their integrity across languages.

## Statistical analysis

The variables were summarized according to their nature. Means and standard deviations were estimated for continuous variables and frequencies for categorical variables. The total amount of sodium intake was calculated using both the 24-hour recall and the reported intake/direct measurement of added salt and seasoning powder. The distribution of dietary and urinary Na and the main variables was verified for normality using the Shapiro–Wilk test. Pearson correlations (for normally distributed data) were applied for the dietary and urine Na. Bland Altman plot was built to see the concordance between both variables (dietary and urine Na), we assessed graphically the assumptions verifying no discernible shape in the graph [19]. Previous publications with small sample sizes (between 15 and 30) used this same method to evaluate concordance [20]. Linear regression models were run in order to estimate the contribution of each NOVA group Na from salt and from seasoning powder to total dietary Na, using the post-estimation Shapley value decomposition, in order to assess the relative contributions of each predictor variable (percentage of contribution to R^2^) on the total Na intake. For the linear regression model, the assumption of constant variance was tested with the Breusch–Pagan/Cook–Weisberg, and normality graphing the the residuals. Finally, a fixed-effect longitudinal regression model was used to estimate the change in NOVA consumption between the initial and final measurements within subjects, with NOVA 1 as the reference group. The assumptions of the models and the distribution were assessed. All statistics were performed using Stata 17 (Stata corp).

## Results

In total there were 35 participants that ranged in age from 14 to 65 years, with an average age of 44 (± 13.1) years. Of the participants, 91.4% were women primarily responsible for food preparation in the household. The families of the participants varied in number of members, ranging from 1 to 12 members, with 43% of the families reporting having 3 to 4 members. Regarding non-communicable diseases in the family, the most prevalent was overweight and obesity (82%), followed by hypertension (40%) and type 2 diabetes (23%). These prevalences were calculated over total households. Most of the participants purchased their groceries at the local street market (“tianguis”) as it is the most accessible and affordable option for them, followed by the supermarket and the small neighborhood store.

The general questionnaire revealed that 82.86% of participants fall within the low socioeconomic status category, 11.43% in the middle-lower and 5.71% in the medium-high. All participants reported being aware that excessive salt intake increases health risks. However, only 68.5% associated it with high blood pressure, and 20% associated it with kidney disease. Furthermore, 91.4% indicated that salt is essential or very important to them as a flavor enhancer, however, 88.5% do try to reduce their salt intake, primarily by not adding more salt after cooking their meals. Additionally, 25% attempted to reduce their consumption of processed foods, 8.5% checked the salt/sodium content in food products, 17% did not add salt while cooking and instead used spices to flavor their dishes, and 14% avoided eating out.

For the direct measurement, visits were made to five of the families’ nearest households of Centro Meneses and fifteen families responded to the online questionnaire. On average, the added salt contributed 509 mg of Na per day, while the seasoning powder contributed 1.59 mg of Na per day, contributing to 10% of the total dietary Na intake.

In those same visits and online questionnaire, the home food inventory was administered. It was observed and documented at household level that more than half of the foods found in the homes belonged to group 1 of the *NOVA classification*, which includes minimally processed foods, while only 10% belonged to NOVA group 4 or Ultra-Processed Foods (UPF). The most commonly found items were beans, carrots, chicken, cucumber, fresh fruit, milk, beef or pork and fresh vegetables (NOVA group 1), followed by table salt and oil (NOVA group 2), regular cheese and cream (NOVA group 3) and lastly by white bread or rolls, ketchup, mustard and mayonnaise (NOVA group 4).

After obtaining the results of the 24 h using ASA24, all the foods and beverages were categorized according to the NOVA classification to determine their degree of processing [13]. Contrasting to the family level, at individual level 26% were UPF (NOVA group 4), 29% processed, 43% raw or natural foods and the rest ingredients in the first recall. In the post intervention the UPF decreased to 20% and the natural foods to 36%, the processed products increased to 42% and the ingredients stayed the same proportion. The results of the estimation of change in NOVA consumption between initial and final measurements within subjects with NOVA 1 as reference group, show that, in general, consumption decreased by 1% (NOVA 2 β = −1.00, 95% CI −1.22, −0.78; NOVA 3 β = −0.97, 95% CI −1.07, −0.87; NOVA 4 β = −1.01 95% CI −1.14, −0.88).

**Fig. 3:**
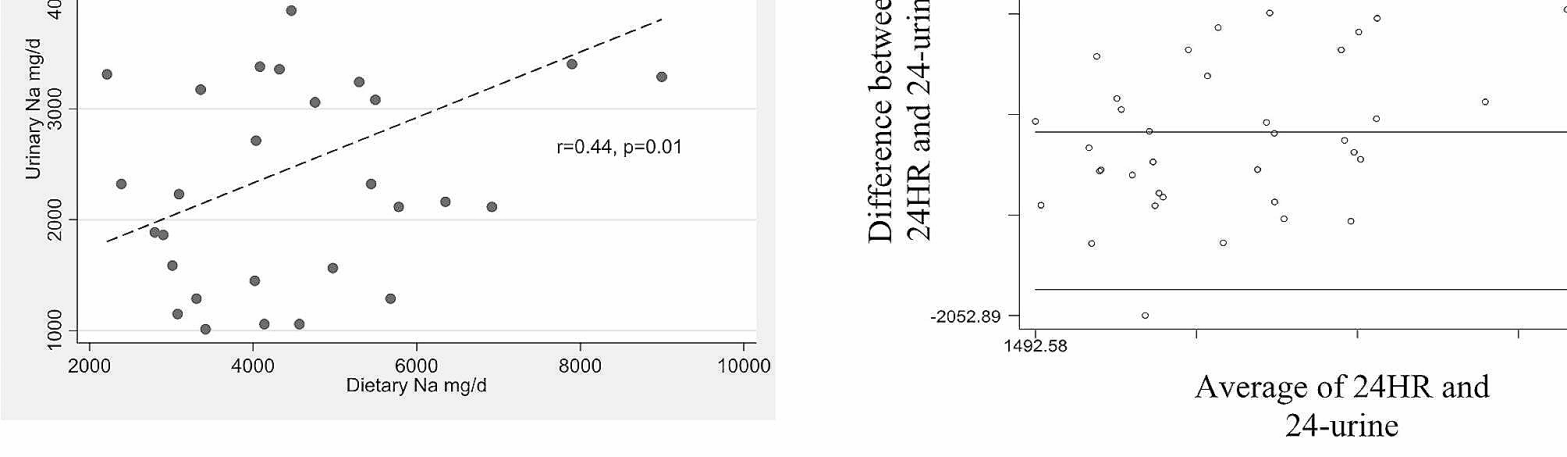
Correlation and concordance between Dietary Na (24 h) and Na in urine

The 24 h method was used to determine the participants’ Na consumption because it has been found to have the best correlation with the 24-hour urine collection (*r* = 0.44, *p* = 0.01) (Fig. 3).

After three months of the intervention, there was a mean reduction of 568 kcal (SD = 775.53 kcal) from the initial measurement (*n* = 31) to the final (*n* = 11) in the 24-hour recall, as well as a reduction in sodium intake of 976 mg (SD = 295.94 mg) (Table 1).

**Table 1:** Comparison of the 24 h in baseline and final measure

Variable	Unit	Baseline measurement *n* = 31				Final measurement *n* = 11				p value
		Mean	SD	Min	Max	Mean	SD	Min	Max	
Energy	kcal	2453.1	944.8	931.6	4800.0	1885.5	490.1	1092.4	2488.0	0.06
Sodium	mg	4221.0	1753.6	1259.1	8995.8	3245.2	1439.4	1567.0	5776.0	0.05
Potasium	mg	3500.3	1347.0	1155.5	7132.2	2570.0	585.0	1954.9	3624.04	0.03

Paired t-test for group mean comparison

Out of the 35 participants who completed the general questionnaire, 31 provided their urine samples for the first measurement, which showed an average concentrated excretion of 2547 mg of Na and 1561 mg of K. For the final measurement, 11 participants provided a urine sample, and there was an average reduction in Na of 325 mg (Table 2).

**Table 2:** Baseline vs. final measurement of 24-hour urine samples

Variable	Unit	Baseline measurement *n* = 31				Final measurement *n* = 11				p value
		Mean	SD	Min	Max	Mean	SD	Min	Max	
24-hour urine volume	mL	1487.74	715.63	450	3040	1405.46	581.56	625	2475	0.36
Density	–	1.02	0.01	1.01	1.03	1.02	0.01	1.01	1.03	0.37
Relative conc. of K in urine	mg	1558.24	696.99	557.7	3623.1	1229.21	722.18	503.1	2850.9	0.09
Relative conc. of Na in urine	mg	2547.07	1142.8	1012	4968	2222.64	1425.49	460	5290	0.22

Paired t-test for group mean comparison

Finally, the linear regression model estimated that for each UPF (NOVA group 4) item reported by the participant in the 24-hour recall, there was an average increase of 80 mg of Na (*p* = 0.01) in the diet compared to minimally processed foods (NOVA group 1), and its contribution was half of the total Na consumption. Similarly, for each processed food (NOVA group 3) there was an average increase of 89.4 mg of Na (*p* = 0.03) in the diet compared to minimally processed foods (NOVA group 1). To determine the percentage of Na from each food group, as well as from salt and/or seasoning powder, after running the regression, the Shapley value was calculated (Table 3). This value indicated that the variable that most predicts Na in the diet was the consumption of ultra-processed foods (Nova group 4), followed by NOVA group 3 or processed foods.

**Table 3:** Regression model of the contribution of NOVA groups to total Na consumption

	Coef	p value	95% IC		Shapley value Percent estimate
NOVA group 1	Reference				
NOVA group 2	−57.3	0.52	−237.3	122.7	4%
**NOVA group 3**	**89.4**	**0.03**	**9.5**	**169.4**	**33%**
**NOVA group 4**	**80.9**	**0.01**	**18.2**	**143.6**	**53%**
Na from seasoning powder	−122.0	0.49	−479.7	235.8	2%
Na from salt shaker	1.1	0.18	−0.5	2.7	8%

In relation to the cooking workshops, the maximum number of participants who joined was 16 persons, with an average attendance of 9 participants in the 7 workshops, resulting in a participation compliance rate of 56%. Also, participants sent a photo of the finished dish. The workshops were recorded, and the recording link was shared with participants who couldn’t attend or wanted to review the workshop. On the other hand, there were 3 gardening workshops, with a maximum attendance of 6 participants. The compliance rate was 83%, and like in the cooking workshops, the recording was sent to them after completion. Some participants sent pictures of their gardens.

Regarding the qualitative data, the following testimonials were obtained about the project:

1. *“This project has been a life-changer for me. I have learned so many new recipes that are easy to make and affordable. It has allowed me to acquire new skills and tools, and I have made significant changes in my lifestyle. I used to have hypertension, but now my blood pressure is normal. I have incorporated new dishes into my diet, and having my own family garden has been a tremendous help both financially and emotionally. I am truly grateful for the impact this project has had on my life.”*
2. *“I really enjoyed the cooking workshops. The recipes were delicious, and I couldn’t stop eating the salads. They are so flavorful, and I don’t even miss the salt. The combination of basil, cilantro, oil, and lemon gives such a wonderful taste. Thank you so much for the recipe!”*
3. *“I am very thankful because the recipe turned out delicious. I paired it with a corn tortilla, and it was a delightful meal. I am sharing a photo of it with you. I might have gone a bit overboard with the rice, but I wanted to make the most of it. It truly tastes amazing. Thank you for providing the recipe.”*
4. *“Hello, good morning. I couldn’t attend last Wednesday’s session, but I made my salad today, and it turned out great. It was super delicious.”*
5. *“It’s DELICIOUS! It turned out so tasty. I like it better like this, more natural.”*

Based on the testimonials, it is evident that the project has been perceived as positive by the participants. The testimonials highlight transformative experiences, ranging from lifestyle changes and improved health to the acquisition of new culinary skills.

## Discussion

This study aimed to address the challenges of urbanization, dietary patterns, and excessive sodium intake in a country with the highest rates of hypertension and cardiovascular diseases by implementing family gardens with aromatic herbs. We showed that the combined interventions (garden materials, workshops, book recipe) improved dietary habits.

To our knowledge this is the first community intervention using a combination of a family garden with aromatic herbs and training (workshops) to reduce Na intake and excretion. The 3-month intensive intervention showed that the involvement of the family on the program can significantly decrease the Na intake. Also, the participants decreased the amount of UPF and substitute them by processed foods at the end of the program, and there was a positive attitude towards healthy lifestyle. Our results are aligned with previous studies that showed a benefit of community gardens on chronic and non-communicable disease through physical activity, improved nutrition and reduced stress [21, 22].

One of the strengths of this project is that we are able to measure Na in 24-hour urine sample, which is considered the “gold standard” for estimating sodium intake in diets [23]. Previous studies in healthy individuals have reported that 92.8% of ingested sodium is excreted in the 24-hour urine [24]. We confirmed that the dietetic method that presented a better agreement with the Na in 24-hour urine was the 24 H-recall, as we showed in the Bland Altman plot [25]. This result is important for future studies in order to account Na intake through the 24 H-recall in bigger samples, as it is not always feasible to collect 24-hour urine in large samples.

One important result to highlight was that, in this population, seasoning powder is not commonly used for cooking, only seven out of the twenty-two participants reporting that they add seasoning powder to their food. So we expected that in population with a higher use of seasoning powder the impact of having an herb family garden could be more, as aromatic herbs replace better the powders as shown in previous publications [26].

We also confirmed that, in a low socioeconomic setting, the combination of interventions (garden materials, workshops, book recipe) motivates the participants to start gardening and use the herbs when preparing meals. The involvement with other families in the workshops increases also the motivation of the participants and facilitate the transmission of knowledge between them.

It is important to emphasize that the project was carried out during the COVID-19 pandemic. The sanitary measures implemented to control the pandemic led to a halt in social activities. Therefore, it was essential to plan remote activities and project actions to ensure the success of the proposed objectives. Furthermore, the pandemic posed a significant threat to public health, emphasizing the need for individuals to prioritize their well-being. Although most workshops were held remotely, we had good compliance (above 50%) and participation. The design and delivery of this intervention also could be a reference to use media and video calls tools and get the information to remote communities who are the most vulnerable and present a high prevalence of non-communicable diseases.

Lastly, the cooking workshops and herb gardening components of the intervention appear to have played a synergistic role in promoting general dietary changes and reducing sodium intake among the study participants. By providing practical skills, knowledge, and a sense of community, the cooking workshops empowered participants to make informed choices about their food intake. Additionally, herb gardening fostered a connection to fresh, wholesome ingredients and reinforced the participants’ commitment to healthy living. These findings highlight the importance of comprehensive interventions that address multiple aspects of lifestyle, including cooking skills, self-efficacy, and food awareness, to promote sustainable dietary change.

The study demonstrates strong internal validity through participative families and though families that had more health problems. The results suggest that this type of intervention could be effective for families with similar characteristics. However, considerations must be made regarding the external validity, emphasizing the need for caution when generalizing findings beyond the specific community and demographic characteristics described, specifically because of the small sample size and the sampling method. Nevertheless, variables such as climatic adaptation to family herb gardens, could be replicated in other studies made in the same area with the same microclimate.

Mexico City is a metropolitan area with almost 10 million inhabitants [27]. Given that at least half of them share similar sociodemographic conditions as the participants in our project [28], replicating this project in other neighborhoods would be promising. We have proven that this intervention is cost-effective and scalable. The detailed description of the intervention in this manuscript, along with all the information generated (including the book recipe, workshops, and questionnaires), can serve as an innovative and feasible approach for decision-makers and other public health professionals to reduce Na intake and, in the long term, decrease hypertension.

This Project is aligned with the Sustainable Development Goals, specifically aim 3 *“Ensure healthy lives and promote well-being for all at all ages”*. In the context of big cities with crowded spaces, having a small garden at home, producing plants that can be used for cooking and giving flavor to food with no added salt or seasoning powder, represents a big opportunity for families to improve diet, particularly dietary habits associated with hypertension.

### Electronic supplementary material

Below is the link to the electronic supplementary material.

Supplementary Material 1

## Data Availability

The datasets and materials used or analyzed during the current study are available from the corresponding author upon reasonable request. Researchers interested in accessing the data and materials should contact Alejandra Cantoral at alejandra.cantoral@ibero.mx. The information will be provided in a format that ensures confidentiality and compliance with ethical and privacy considerations.
